# Impaired immune and coagulation systems may be early risk factors for COVID-19 patients: A retrospective study of 118 inpatients from Wuhan, China

**DOI:** 10.1097/MD.0000000000021700

**Published:** 2020-08-28

**Authors:** Zhi-jun Qin, Lei Liu, Qun Sun, Xia Li, Jian-fei Luo, Jia-sheng Liu, Dan Liu

**Affiliations:** a Department of Intensive Care Unit; b Department of Infection Management, Sichuan Provincial Orthopedic Hospital, Chengdu, Sichuan; c Department of Gastrointestinal Surgery, Renmin Hospital of Wuhan University, Wuhan, Hubei; d Department of Respiratory and Critical Care Medicine, West China Hospital of Sichuan University, Chengdu, China.

**Keywords:** Coronavirus Disease 2019, logistic regression, ROC curve, hospital mortality

## Abstract

The coronavirus disease 2019 (COVID-19) outbreak has become a global health threat and will likely be one of the greatest global challenges in the near future. The battle between clinicians and the COVID-19 outbreak may be a “protracted war.”

The objective of this study was to investigate the risk factors for in-hospital mortality in patients with COVID-19, so as to provide a reference for the early diagnosis and treatment.

This study retrospectively enrolled 118 patients diagnosed with COVID-19, who were admitted to Eastern District of Renmin Hospital of Wuhan University from February 04, 2020 to March 04, 2020. The demographics and laboratory data were collected and compared between survivors and nonsurvivors. The risk factors of in-hospital mortality were explored by univariable and multivariable logistic regression to construct a clinical prediction model, the prediction efficiency of which was verified by receiver-operating characteristic (ROC) curve.

A total of 118 patients (49 males and 69 females) were included in this study; the results revealed that the following factors associated with in-hospital mortality: older age (odds ratio [OR] 1.175, 95% confidence interval [CI] 1.073–1.287, *P* = .001), neutrophil count greater than 6.3 × 10^9^ cells/L (OR 7.174, (95% CI 2.295–22.432, *P* = .001), lymphocytopenia (OR 0.069, 95% CI 0.007–0.722, *P* = .026), prothrombin time >13 seconds (OR 11.869, 95% CI 1.433–98.278, *P* = .022), d-dimer >1 mg/L (OR 22.811, 95% CI 2.224–233.910, *P* = .008) and procalcitonin (PCT) >0.1 ng/mL (OR 23.022, 95% CI 3.108–170.532, *P* = .002). The area under the ROC curve (AUC) of the above indicators for predicting in-hospital mortality were 0.808 (95% CI 0.715–0.901), 0.809 (95% CI 0.710–0.907), 0.811 (95% CI 0.724–0.898), 0.745 (95% CI 0.643–0.847), 0.872 (95% CI 0.804–0.940), 0.881 (95% CI 0.809–0.953), respectively. The AUC of combined diagnosis of these aforementioned factors were 0.992 (95% CI 0.981–1.000).

In conclusion, older age, increased neutrophil count, prothrombin time, d-dimer, PCT, and decreased lymphocyte count at admission were risk factors associated with in-hospital mortality of COVID-19. The prediction model combined of these factors could improve the early identification of mortality risk in COVID-19 patients.

## 1 Introduction

In December 2019, a new type of acute respiratory infectious disease occurred in Wuhan, Hubei, China,^[1]^ which was named as “coronavirus disease 2019 (COVID-19)” on February 11, 2020 by the World Health Organization (WHO).^[2]^ On March 11, WHO alerted that COVID-19 could be characterized as a pandemic disease,^[3]^ and the alarm bell was rung loudly and clearly. As of April 10, SARS-CoV-2 has spread to >210 countries, with a cumulative total of 1.5 million confirmed cases and >90,000 deaths.^[4]^ Early identification of disease risk factors is the basis of clinical treatment, and many scholars have conducted a lot of exploration in the early stage of the disease outbreak. Although the mortality of COVID-19 varies markedly among different countries and regions, patients with preexisting hypertension, diabetes, coronary heart disease, and other chronic diseases have worse clinical outcomes, and older age, higher SOFA score, and elevated d-dimer at admission were considered risk factors for in-hospital mortality of COVID-19 patients.^[5,6]^ The aim of the study was to re-identify the risk factors and to develop a clinical prediction model for predicting in-hospital mortality in COVID-19 patients

## 2 Patients and methods

### 2.1 Study design and population

This retrospective study was conducted in the Eastern District of Renmin Hospital of Wuhan University, which was a government-designated institution for the treatment of severe COVID-19 in Wuhan. We retrospectively reviewed the electronic medical record of the patients who were admitted from February 04, 2020 to March 04, 2020 and had been discharged or died during this period. All patients were laboratory confirmed COVID-19 cases, which met the diagnostic criteria for confirmed cases of the COVID-19 Diagnosis and Treatment Plan (Trial Seventh Edition) ^[7]^ issued by the National Health Commission of the People's Republic of China. Patients were excluded based on the following criteria: patients younger than 18 years; time from illness onset to hospital admission exceeded 14 days; pregnant or parturient; patients transferred to the “mobile cabin hospital” during hospitalization. Patients were classified as survivors and nonsurvivors based on clinical outcome (discharge or death).

The study was approved by Clinical Trial Ethics Committee of Renmin Hospital of Wuhan University (WDRY2020–K068) and the requirement for informed consent was waived by the Ethics Commission.

### 2.2 Data collection

Demographic, clinical, laboratory testing, treatment, and outcome data were extracted from electronic medical records including the information of demographic data, current medical history, comorbidities, symptoms and signs, laboratory parameters, respiratory support mode, and clinical outcomes. All information were double-checked by 2 researchers (Z-jQ, QS).

### 2.3 Statistical analysis

Continuous and categorical variables were presented as median (interquartile range) and n (%), respectively. The Mann–Whitney *U* test and *χ*^2^ test were used to compare the difference between survivors and nonsurvivors. To explore the risk factors associated with in-hospital death, univariable and multivariable logistic regression models were used to screen the potential predictors. Univariate analysis was performed at the first step, and statistically significant variables were included in the multivariate stepwise regression analysis. To verify the predictive effectiveness of the regression model, the ROC curves were drawn and compared with the individual factors.

## 3 Results

Of the 247 patients admitted from February 04, 2020 to March 04, 2020 and had been discharged or died during this period, 129 patients were excluded based on the following reasons: 1 patient was younger than 18 years, 69 patients had symptom over 14 days from illness onset to hospital admission, 49 patients were transferred to the “mobile cabin hospital” during hospitalization, and 10 patients had COVID-19 infection before delivery. A total of 118 patients were included in the final analysis, with 77 survivors and 41 nonsurvivors.

### 3.1 Univariate analysis for COVID-19

Mean age of 118 patients (49 males and 69 females) was 63.1 ± 15.7 years. Mean age of nonsurvivors was significantly older than that of survivors (73.8 ± 13.1 vs 57.4 ± 13.9 years) (*P* < .001). Nonsurvivors had higher frequencies of hypertension, coronary heart disease, and chronic kidney disease than that of survivors (*P* = .001, *P* = .021, and *P* = .005, respectively). There were higher APACHE**II** scores, more intensity of respiratory support and longer duration of mechanical ventilation in nonsurvivors (*P* < .001). Length of hospital stay of nonsurvivors was significantly shorter than that of survivors (*P* < .001).

Laboratory tests showed the following parameters were at a higher level in nonsurvivors compared with survivors: neutrophil count (*P* < .001), C-reactive protein (*P* < .001), prothrombin time (*P* < .001), d-dimer (*P* < .001), PCT (*P* < .001), cardiac troponin I (*P* < .001), creatine kinase (*P* = .041), creatine kinase isoenzyme-MB (*P* < .001), lactate dehydrogenase (*P* < .001), creatinine (*P* < .001), glucose (*P* < .001). And the parameters at a lower levels were as follows: lymphocyte count (*P* < .001), platelet count (*P* < .001), antithrombin III (*P* < .001), albumin (*P* < .001) (Table 1).

**Table 1:**
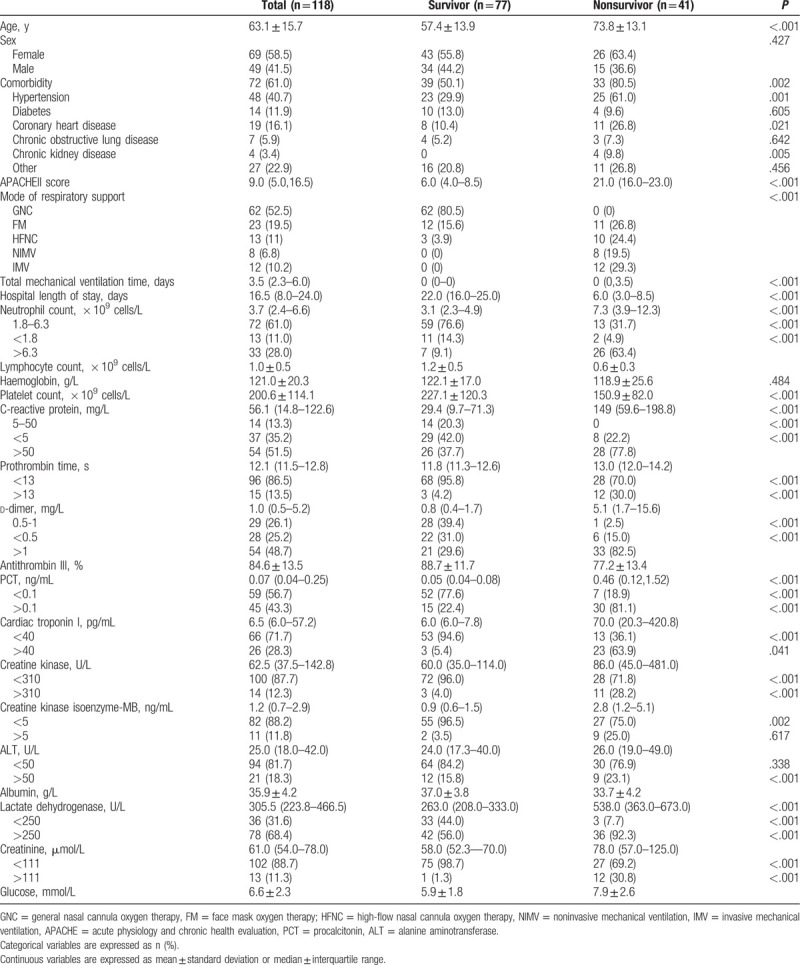
Demographics and laboratory data at admission in the patients with COVID-19.

### 3.2 Multivariable regression analysis for COVID-19

Multivariable logistic regression showed that older age, neutrophil count >6.3 × 10^9^ cells/L, lymphocytopenia, prothrombin time >13 seconds, d-dimer >1 mg/L, and PCT >0.1 ng/mL at admission were risk factors associated with in-hospital mortality (OR 1.175, 95% CI 1.073–1.287, *P* = .001; OR 7.174, 95% CI 2.295–22.432, *P* = .001; OR 0.069, 95% CI 0.007–0.722, *P* = .026; OR 11.869, 95% CI 1.433–98.278, *P* = .022; OR 22.811, 95% CI 2.224–233.910, *P* = .008 and OR 23.022, 95% CI 3.108–170.532, *P* = .002, respectively, Table 2).

**Table 2:**
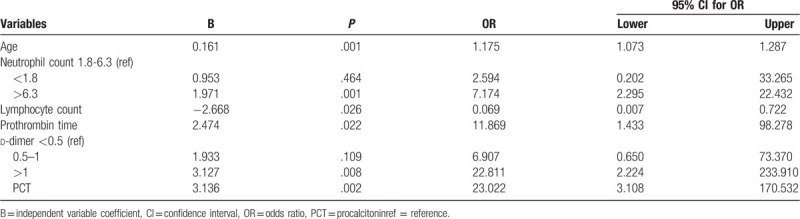
Risk factors for the in-hospital mortality of COVID-19 by multivariable logistic regression.

### 3.3 ROC curve analysis

**Figure 1:**
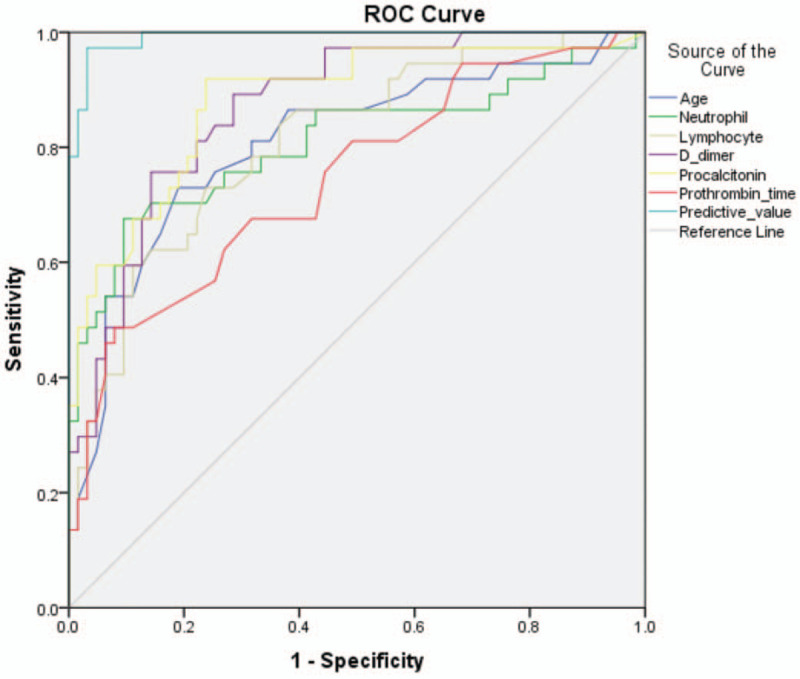
Age, neutrophil count, lymphocyte count, prothrombin time, d-dimer and PCT at admission in predicting the in-hospital mortality of COVID-19 by ROC analysis. The independent AUC value of the above six factors predicting in-hospital mortality was from 0.745 to 0.881, whereas the AUC value of these combined factors rose to 0.992. AUC = area under the curve, ROC = receiver-operating characteristic, PCT = procalcitonin.

The AUC values of age, neutrophil count, lymphocyte count, prothrombin time, d-dimer and PCT for predicting the in-hospital mortality of COVID-19 patients were 0.808 (95% CI 0.715–0.901), 0.809 (95% CI 0.710–0.907), 0.811 (95% CI 0.724–0.898), 0.745 (95% CI 0.643–0.847), 0.872 (95% CI 0.804–0.940), 0.881 (95% CI 0.809–0.953 (Fig. 1), respectively. While combining the bundle of risk factors selected by logistic regression, the AUC value rose to 0.992 (95% CI 0.981–1.000) (Table 3, Fig. 1).

**Table 3:**
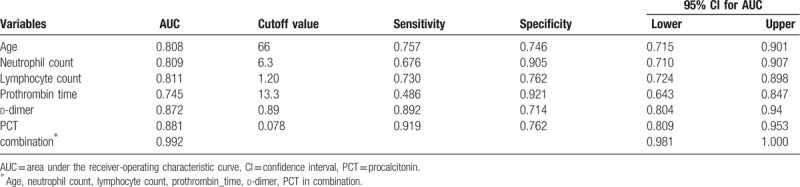
Predictive values of parameters for the in-hospital mortality of COVID-19.

## 4 Discussion

In this study, some objective parameters of patients at admission were taken as variables. Similar to previous findings, nonsurvivors were older and had more comorbidities including hypertension, coronary heart disease, and chronic kidney disease.^[6,7]^ In addition to older age, increased neutrophil count, prothrombin time, d-dimer, PCT and decreased lymphocyte count were detected be risk factors for in-hospital mortality in COVID-19. We speculate that impairment of the immune and coagulation systems based on the hyper-inflammatory response may be the early dangerous signs for COVID-19 patients.

Higher neutrophil count, C-reactive protein, and decreased lymphocyte count were present in non-survivors, and multiple logistic regression model showed that increased neutrophil count and lymphocytopenia were risk factors for in-hospital mortality in COVID-19. Lymphocytopenia is an early characteristic of COVID-19 patients, which predicts impaired immune system and leads to a decreased ability of the body to clear viruses.^[8–10]^ Damaged immune organs found in autopsy may be the pathological basis of hypolymphocytosis.^[11]^ Neutrophils are key players in cytokine regulation and release of inflammatory mediators, increased neutrophil numbers may be closely related to the development of cytokine storm.^[11,12]^ Based on increased neutrophil count, C-reactive protein and decreased lymphocyte count, the phenomenon of “separation of neutrophils and lymphocytes” appeared, meanwhile the higher neutrophil-to-lymphocyte ratio and lower lymphocyte-to-C-reactive protein ratio were found in nonsurvivors, which reflect an enhanced inflammatory process may suggest a poor prognosis.^[13]^

There were more prolonged prothrombin time, increased d-dimer, reduced antithrombin III activity, and decreased platelet count in nonsurvivors. Prothrombin time >13 seconds and d-dimer >1 mg/L predicted an increased risk of in-hospital mortality in COVID-19. Coagulation disorders in COVID-19 might be associated with factors such as cytokine release syndrome, immune damage of the hematopoietic system, and reperfusion injury.^[14,15]^ As the disease progressed, the microthrombus load caused by the activation of coagulation system rose gradually, whereas the activation and consumption of anticoagulant system, primary/secondary hyperfibrinolysis were more serious, and the bleeding tendency was more severe. These pathological changes may happen continuously and alternately at different stages of the disease, which were consistent with the process of diffuse intravascular coagulation in critically ill patients. With the progression of diffuse intravascular coagulation, the patients experienced serious complications such as bleeding and embolism of vital organs, multiple organ dysfunction syndrome, and eventually died.

Clinicians often use PCT to differentiate bacterial infections from systemic inflammatory responses of other etiologies; however, increased PCT is also manifested in various viral infections.^[16,17]^ There are significant differences in PCT levels in infections caused by different viruses, and coronavirus, influenza A, and human rhinovirus/enterovirus infections had higher serum levels of PCT.^[18,19]^ PCT was uncommon to be found increased in previous studies,^[20,21]^ and was also <0.1 ng/mL in most patients in this study. However, patients with increased PCT were significantly associated with death, multiple logistic regression model indicated that PCT >0.1 ng/mL was a risk factor for in-hospital mortality in COVID-19. Undoubtedly, this cutoff value is significantly lower than the predictive value for patients with septic shock due to bacterial infection.^[22,23]^ This suggests that the alert value of PCT for assessing disease prognosis and severity may have to be significantly downregulated in COVID-19 patients. Furthermore, many studies have confirmed that the extent of PCT rise is highly correlated with disease severity,^[17,23–26]^ and similar result was found in our study. Fourteen of 15 patients with PCT >1 ng/mL eventually died and plasma PCT levels were significantly higher in non-survivors than in survivors. The induction amount and the plasma level of PCT directly correlate with the inflammatory reaction,^[27]^ which indicates that enhanced responses caused by SARS-CoV-2 and more severe tissue damage in patients. Intense inflammatory response and tissue damage lead to severe organ dysfunction, and higher cardiac troponin I, creatine kinase, creatine kinase isoenzyme-MB, lactate dehydrogenase, creatinine, and other organ damage indicators occurred in nonsurvivors. However, the same damage is also inevitable in the immune system and coagulation system.

This study has some limitations. First, this was a retrospective single-center study for COVID-19; the potency of the results might be affected by small sample size and partially missing laboratory parameters in some patients. Second, some patients were transferred from other hospitals, and heterogeneous medical intervention before this admission may affect the prognosis of patients. Third, we screened patients based on established clinical outcomes (discharge/death), whereas some patients who admitted during the same period and still hospitalized were not included, so that the proportion of deaths in this study was not representative of actual COVID-19 mortality rate.

## 5 Conclusions

In conclusion, we found that older age, increased neutrophil count, prothrombin time, d-dimer, PCT, and decreased lymphocyte count were predictors associated with in-hospital mortality of COVID-19. Combined diagnosis of these factors can improve the early identification of mortality risk in COVID-19 patients.

## Acknowledgments

We acknowledge all health care workers involved in the diagnosis and treatment of patients and all those who contributed to the fight against the COVID-19 outbreak in Wuhan, and we thank Yi Li, Xi Yang, Tian-zhi Qin for their help in the data collation of this study.

## Author contributions

**Conceptualization:** Zhi-jun Qin, Xia Li, Jia-sheng Liu, Dan Liu.

**Data analysis:** Lei Liu, Xia Li.

**Data collection:** Zhi-jun Qin, Qun Sun.

**Data curation:** Zhi-jun Qin, Qun Sun.

**Formal analysis:** Lei Liu, Xia Li.

**Project administration:** Jia-sheng Liu, Jian-fei Luo, Dan Liu.

**Writing – original draft:** Zhi-jun Qin.

**Writing – review & editing:** Lei Liu, Dan Liu.
